# The learning curve for hood‐sparing robotic‐assisted radical prostatectomy: A single‐surgeon experience

**DOI:** 10.1002/bco2.463

**Published:** 2024-11-18

**Authors:** Keith R. S. Simpson, Jamie Krishnan, Linda Taylor, Alan McNeill, Daniel W. Good

**Affiliations:** ^1^ Urology Department Western General Hospital Edinburgh UK

**Keywords:** hood sparing, learning curve, RARP, robot‐assisted radical prostatectomy, urinary incontinence

## Abstract

**Objectives:**

This study aimed to assess the impact of anterior hood‐sparing robot‐assisted radical prostatectomy (RARP) with posterior‐anterior reconstruction in a single‐surgeon series by analysing oncological and functional continence outcomes.

**Patients and Methods:**

We carried out a cohort comparison study of a prospectively collected single‐surgeon series. The surgeon was an ‘in‐training’ fellowship trained surgeon in their first 2 years of independent practice. There were three cohorts identified from electronic and scanned paper operation notes. The first cohort of standard anterior RARP (no hood sparing) included initial patients and any patient in the consecutive series who had completed 3 month FU after RARP. The second cohort was hemi‐hood‐sparing RARP again within the consecutive database of patients and lastly full‐hood‐sparing RARP. Early continence was defined by patients reporting being ‘dry’ and with 0 pad or 1 confidence/security pad. Data was collected in an Excel spreadsheet, and SPSS was used to assess distribution with non‐parametric data being analysed using a Mann Whitney *U* test and parametric data with an unpaired *t*‐test.

**Results:**

We identified 174 patients from March 2020 to February 2022 who were operated on. Full pathology and 6‐week follow‐up pad use data was available for all patients. At 12 months, some data for EPIC‐26 was not available (lack of response/clinic non‐attendance). The results demonstrate doubling in early continence to over 75% at 6‐week follow‐up with comparable positive margin rates. This difference was statistically significantly better in the dorsal venous complex RARP sparing group in comparison to standard RARP (*p* < 0.001).

**Conclusion:**

Anterior hood‐sparing RARP with anterior reconstruction is a modification to the standard anterior RARP approach with a short learning curve which provides patients with better early and late continence without compromise to oncological outcomes.

## 1 INTRODUCTION

Robot‐assisted radical prostatectomy (RARP) was developed over 20 years ago based on established laparoscopic techniques.^1^ Urinary incontinence is a major side effect of the procedure which significantly impairs patient quality of life.^2^ Recently, focus has turned towards modifications of the surgical technique to improve patient quality of life outcomes without sacrificing oncological control. One such method which has gained popularity in recent years is the Retzius‐sparing approach^3^ which approaches the prostate from the pouch of Douglas, as opposed to the classically described anterior approach.^4^ The Retzius‐sparing approach is felt to be more technically difficult and is associated with reports of higher positive surgical margin (PSM) rates in early Retzius‐sparing studies;^5^ although, the largest meta‐analysis of the Retzius‐sparing learning curve reported no increased likelihood of PSM when compared to standard resections.^6^ In response to these concerns, Cohelo et al. reported on an adaptation of the standard anterior approach for performing prostatectomy whilst preserving anterior neurovascular bundles and other key structures within the space of Retzius.^7^ This approach was later dubbed the ‘hood technique’ (HT) by Wagaskar et al., who also described in detail the various retropubic structures which are preserved.^8^ Early HT case series have shown promising results with respect to both return to continence and PSM rates.^7^, ^8^, ^9^

These same studies have highlighted a need for further study to confirm the generalisability of these findings. To this end, our objective was to assess the impact of anterior hood‐sparing RARP with posterior–anterior reconstruction compared with standard RARP, in a single‐surgeon series with respect to oncological and functional continence outcomes.

The role of learning curve and surgeon experience is emphasised in our study as the cases examined include the surgeon's first hood‐sparing resections. Learning curve has been highlighted as a significant factor which may explain differential PSM rates between these novel approaches,^5^ as the finding of significantly higher PSM rates in early cases after adopting the Retzius‐sparing approach has been limited to less experienced robotic surgeons, with expert surgeons not demonstrating the same issue.^10^

## 2 PATIENTS AND METHODS

We examined a prospectively collected single‐surgeon consecutive series of 174 patients who underwent RARP with either standard anterior approach, bilateral hood‐sparing or ‘hemi’ hood‐sparing resection, between March 2020 and February 2022. Further analysis also grouped patients who underwent either bilateral or hemi‐hood‐sparing resections into an ‘any’ hood‐sparing category. All patients had either T2 or T3 disease. In contrast to many other published series,^7^, ^8^, ^9^ the operating surgeon was in their second year of independent practice, having performed an ‘in‐training’ fellowship. Patients were identified through analysis of paper or scanned operation notes. PROM data were collected prospectively using Redcap® database, and questionnaires emailed to patients at baseline and 3 and 12 months post‐operatively. Data was collated into an Excel spreadsheet. Distribution of the data was assessed using SPSS, with non‐parametric data analysed using Mann Whitney *U* test and parametric data with an unpaired *t*‐test.

Patient selection for a particular approach was based on two factors, timing within the case series and clinical evidence of more locally advanced (radiological T3a/b) or large volume anteriorly located disease. The surgeon had initially been performing only standard anterior technique resections, adopting the HT mid‐way through the series. After that time, the decision to perform a HT resection was made at the surgical planning stage, based on radiological and pathologic data. High‐volume bilateral T3a/b disease was performed with a standard approach to minimize margins. Equally, bilateral T3a anterior disease also necessitated a standard anterior approach. However, the majority of case (unilateral T3a/b) and all T2 disease would be suitable for a hood‐sparing technique. For those high‐volume locally advanced disease (T3a/b) unilateral disease, a hemi‐hood‐sparing technique was employed in order to ensure adequate oncological control.

Pre‐operative parameters included in the analysis were age, American Society of Anesthesiologists (ASA) score, mean pre‐op prostate‐specific antigen (PSA), pathological T stage and baseline EPIC‐26 UI score. The key outcomes were PSM rate, early continence rate (at 6–8 weeks), late continence rate (at 12‐month follow‐up), EPIC‐26 UI score at 6–8 weeks, EPIC 26‐UI score at 12 months, mean operating time, mean blood loss and complication rate. Patients were deemed continent if reporting they were ‘dry’ with either 0 pad or 1 ‘confidence pad’ used per day.

## 3 RESULTS

With regard to pre‐operative case selection, the groups were largely similar. Among the cases analysed, there was no significant difference between groups with respect to the majority of pre‐operative measures, including age (mean range 66–67, *p* = 0.5), ASA (range 2–2, *p* = 0.2), BMI (mean range 28–28, *p* = 0.3), mean pre‐op PSA (mean range 11.4–12.1, *p* = 0.4) and baseline EPIC‐26 UI score (mean range 88–90, *p* = 0.5) (Table 1). The mean prostate weight (g) was not statistically different between all of the groups, suggesting that prostate size was not a determinant of outcome. When looking at the mean case number (number of cases surgeon has performed) between the groups, there was a lower mean case number (less case experience) in the standard technique compared with the any hood‐sparing group (82 vs 128, *p* = <0.01) (Table 1).

**TABLE 1:** Analysis of a single‐surgeon consecutive series comparing hood‐sparing and standard robot‐assisted radical prostatectomy patients.

Parameter	Standard RARP *N* = 88	Hemi‐hood‐sparing RARP *N* = 33	Bilateral hood sparing *N* = 53	Any hood sparing *N* = 86	*p* value
Age	67 (55–79)	66 (54–77)	66.4 (50–76)	66 (50–77)	0.5^±^
ASA (median)	2 (2–2)	2 (2–2)	2 (2–2)	2 (2–2)	0.2^±^
BMI	28 (19–42)	28 (21–34)	28 (19–36)	28 (19–36)	0.3^±^
Mean pre‐op PSA	11.4 (3.2–52)	12.1 (4–28)	11.9 (3.1–51)	12.0 (3.1–51)	0.4^±^
pT stage					
pT2	29 (33%)	13 (39%)	33 (62%)	46 (53%)	<0.01^¥^
pT3	59 (67%)	20 (61%)	20 (38%)	40 (47%)	0.01^¥^
Positive surgical margin					
Overall	27/88 = 30%	8/33 = 24%	15/53 = 28%	23/86 = 27%	0.5^¥^
pT2	7/29 = 24%	1/13 = 7%	6/33 = 18%	7/46 = 15%	0.1^¥^
pT3 (a/b)	20/59 = 34%	7/20 = 35%	9/20 = 45%	16/40 = 40%	0.6^¥^
% focal margins (<3 mm)	19/27 = 70%	4/8 = 50%	9/15 = 60%	13/23 = 57%	0.1^¥^
Mea prostate weight (g)	53 (22–210)	54 (24–195)	49 (16–98)	51 (16–195)	0.16^±^
EPIC‐26 UI at baseline	88 (54–100)	90 (20–100)	90 (48–100)	90 (20–100)	0.5^±^
Missing data	55%	18%	28%	24%	
EPIC‐26 UI at 12 months	83 (37–100)	89 (62–100)	88 (56–100)	88 (56–100)	0.2^±^, *
Missing data	83%	33%	60%	51%	
EPIC‐26 UI at 6–8 weeks	66 (6–100)	77 (39–100)	79 (58–100)	79 (39–100)	<0.01^±^, *
No data	48%	31%	40%	35%	
Early continence (at 6–8 weeks)	38/88 = 43%	25/33 = 76%	40/53 = 75%	65/86 = 76%	<0.01^¥^
12‐month continence	72/88 = 82%	32/33 = 97%	52/53 = 98%	84/86 = 97%	<0.01^¥^
Median length of hospital stay (day)	1	1	1	1	0.8^±^
Mean operative time (min)—skin to skin	154 (105–270)	148 (110–230)	137 (90–200)	142 (90–230)	0.02^±^
Mean blood loss (mL)	182 (5–1200)	99 (5–850)	74 (0–210)	84 (0–850)	<0.01^±^
Blood transfusions (%)	<1%	0%	1.6%	<1%	
Complication rate (%)	6 (6.8%)	3 (9%)	5 (9%)	8 (8.1%)	0.7^¥^
CD 1–2	4 (4.5%)	2 (6%)	3 (5.6%)	5 (5.8%)	0.6^¥^
CD 3+	2 (2.3%)	1 (<1%)	1 (1.8%)	2 (2.3%)	1.0^¥^

Abbreviations: ASA, American Society of Anesthesiologists; BMI, body mass index; PSA, prostate‐specific antigen; RARP, robot‐assisted radical prostatectomy.

Legend:

^¥^ Chi‐squared test.

^±^ Mann Whitney *U* test.

* Minimum clinically meaningful difference for EPIC‐26 UI described to be 6–9.

There was difference noted in pathological T stage between groups with the standard and hemi‐hood‐sparing groups containing a lower percentage of T2 disease at 33% and 39% respectively compared with 62% of the bilateral hood‐sparing group (*p* < 0.01). Regardless of disease stage, there was no significant difference observed in PSM rates between groups (over all range 24%–30%, *p* = 0.5), (T2 7%–24% range, *p* = 0.1) and (T3 34%–45% range, *p* = 0.6) (Table 1).

Both patient continence and quality of life indicators were higher in the hood‐sparing patients. At the early 6 to 8‐week time‐point, 75%–76% of patients in the hood‐sparing group were continent, compared with only 43% in the standard group (*p* < 0.01). At 1 year, this relationship was still observable with continence rates of 97%–98% in the hood‐sparing cohort vs 82% in the standard cohort (*p* < 0.01). Whilst there was no difference observed, no significant difference between the two groups noted for EPIC‐26 UI score at 12 months (range 83%–89%, *p* = 0.2); hood‐sparing resections had a significantly higher score at 6–8 weeks (66 vs. 77–79, *p* < 0.01) (Table 1). Due to a technical issue with the Redcap server (blocking sending to certain patient email addresses) used for our PROM database, this meant that there was a reduction in the rate of returns for this data.

A post hoc power calculation was performed with an alpha of 0.05; this showed a power of 0.88, indicating that the study was adequately powered to detect a difference in continence outcomes.

Secondary quality indicators were also favourable of the hood‐sparing approach. Mean operative time (137–148 min vs. 154 min, *p* = 0.02) and mean blood loss (74–99 mL vs. 182 mL, *p* < 0.01) were lower with the hood‐sparing approach. Furthermore, no difference was seen between groups with regard to length of hospital stay (median range 1–1, *p* = 0.8) and complication rate (range 6.8%–9%, *p* = 0.7) (Table 1).

## 4 DISCUSSION

This study demonstrated a statistically significant and clinically meaningful improvement in continence rates in hood‐sparing RARP patients at both 6–8 weeks, as well as at 1‐year follow‐up. The hood‐sparing cohort also had superior quality of life scores (EPIC UI) at 1 year when compared to standard RARP patients. This finding is of interest as many other studies of both hood‐sparing and Retzius‐sparing techniques report only improvements in early continence, with later post‐operative data comparable to standard resection.^5^, ^7^, ^11^ Interestingly, this was achievable for an early career surgeon and did not require a long/any learning curve to achieve these results. This suggests that the hood‐sparing approach may be particularly well suited to optimising functional patient outcomes during the RARP learning curve.

Adopting the hood‐sparing technique also resulted in improvements in secondary indicators of surgical quality, with reduced blood loss and faster operations. As would be expected, dissection under the dorsal venous complex results in a plane that leads to lower bloodloss.^6^ The hood‐sparing technique results in a simplified operation. Given the context of our study, these findings indicate that the hood‐sparing resection is a superior approach even for the early‐career surgeon.

Some limitations were identified with the analysis. Firstly, the improved bilateral hood‐sparing results could relate to selection bias as evidenced in a slightly greater proportion of patients with T2 disease (pT2 in 33% standard vs 53% in hood‐sparing group). Secondly, as the early‐career surgeon was initially performing standard‐RARP and began hood‐sparing techniques mid‐sequence, there will be a relative difference in over‐all surgeon experience between the hood‐sparing and standard groups, which could account for some of the differences seen. However, the fact that these improvements in outcome were seen immediately after the change in technique, this is unlikely to account for the observed effect. The learning curve of Retzius‐sparing RARP has been published on previously^10^, ^12^, ^13^, and this was estimated at around 130 cases in the hands of an already very experienced robotic prostate surgeon. In research by Hussein H. and colleagues, their first cohort of patients had a relatively higher T2 PSM rate in comparison to our study where the T2 PSM was below 20%. This suggests an easier adoption of the ‘hood‐sparing’ technique in comparison to Retzius‐sparing RARP. Some might argue the PSM rate of 15% is on the high side; however, snapshot studies have shown that the average T2 PSM rate among UK Urologists was around 17.5% and in high‐volume robotic surgeons 14%.^14^, ^15^ Notably, in our study, adoption of the hood‐sparing technique led to a reduction in the T2 PSM rate to a level similar to the reported UK average. It is worth noting that the largest study of Retzius‐sparing technique did not show an association with PSM in experienced surgeons.^16^

There is controversy as to the best definition for return of continence following radical prostatectomy. Many series use the 0 or 1 confidence pad cut off.^10^, ^12^, ^13^, ^15^ We decided on the use of this definition in order to aid comparison to the majority of the published literature on Retzius‐sparing RARP which is the most common modification of the standard anterior approach which has gained popularity. The long term (1 year) continence outcomes in our hood‐sparing technique is very similar to that published by Galfano et al.^10^ which quoted 96% ‘continence’ for Retzius‐sparing RARP vs 97% for hood‐sparing RARP. It is reassuring that the large prospective^17^ and systematic review and meta‐analyses on the subject have also shown that long‐term continence (1 year) is not different between Retzius‐sparing RARP compared to the anterior technique and only early continence is superior.^18^ The early continence rate in our hood‐sparing RARP were slightly lower than reported in the Retzius‐sparing RARP literature, but the age and average PSA in our series was higher, which may partly explain this difference (77% vs 90% continence at 3 months) as higher age is known to be associated with slower recovery of continence.^19^

The effect of nerve sparing resection was not specifically examined in this analysis. Whilst it was noted that the bilateral hood‐sparing cohort had a higher percentage of T2 disease, and are therefore more likely to have undergone nerve sparing procedures, the improved outcomes were also observed in the hemi‐hood‐sparing group which was not significantly different from the standard RARP group in this regard.

Reassuringly, the peri‐operative outcomes (blood loss, operative time and complication rates) were not significantly different with the hood‐sparing technique reported in our series compared with the published literature.^10^, ^13^, ^17^, ^20^, ^21^ All of this suggests that adoption of the hood‐sparing RARP technique is safe and quick and provides patients with similarly impressive early and long‐term continence without a significant increase in PSM.

## 5 CONCLUSION

Anterior hood‐sparing RARP with anterior reconstruction is a modification to the standard anterior RARP approach with a very short learning curve which provides patients with better early and late continence without compromise to oncological outcomes. Given the advantages of this approach, and the trend of operating on increasingly more aggressive disease, it will be of interest to surgeons wanting to improve their functional outcomes whilst minimising the impact on oncological outcomes.

## AUTHOR CONTRIBUTIONS

Mr Simpson did the data collection, performed the analysis and wrote the paper. Mr Krishnan helped with data collection, critical review and writing of the paper. L. Taylor helped with data collection, critical analysis and writing of the paper. Prof McNeill helped with study design, critical analysis of the data and writing of the paper.

## CONFLICT OF INTEREST STATEMENT

The authors declare no conflicts of interest.

## References

[1] Tewari A , Peabody J , Sarle R , Balakrishnan G , Hemal A , Shrivastava A , et al. Technique of da Vinci robot‐assisted anatomic radical prostatectomy. Urology. 2002;60(4):569–572. 10.1016/s0090-4295(02)01852-6 12385908

[2] Ficarra V , Novara G , Rosen RC , Artibani W , Carroll PR , Costello A , et al. Systematic review and meta‐analysis of studies reporting urinary continence recovery after robot‐assisted radical prostatectomy. Eur Urol. 2012;62(3):405–417. 10.1016/j.eururo.2012.05.045 22749852

[3] Secco S , Galfano A , Barbieri M , Piccinelli M , di D , Napoli G , et al. Technical features and the demonstrated advantages of the Retzius sparing robotic prostatectomy. Arch Esp Urol. 2019;72(3):247–256. 30945651

[4] Berryhill R , Jhaveri J , Yadav R , Leung R , Rao S , el‐Hakim A , et al. Robotic prostatectomy: a review of outcomes compared with laparoscopic and open approaches. Urology. 2008;72(1):15–23. 10.1016/j.urology.2007.12.038 18436288

[5] Stonier T , Simson N , Davis J , Challacombe B . Retzius‐sparing robot‐assisted radical prostatectomy (RS‐RARP) vs standard RARP: it's time for critical appraisal. BJU Int. 2019;123(1):5–7. 10.1111/bju.14468 29959814

[6] Checcucci E , Veccia A , Fiori C , Amparore D , Manfredi M , di Dio M , et al. Retzius‐sparing robot‐assisted radical prostatectomy vs the standard approach: a systematic review and analysis of comparative outcomes. BJU Int. 2020;125(1):8–16. 10.1111/bju.14887 31373142

[7] de P , Barbosa JABA , Guglielmetti GB , Cordeiro M , Rocco B , Nahas W , et al. Retrograde release of the neurovascular bundle with preservation of dorsal venous complex during robot‐assisted radical prostatectomy: optimizing functional outcomes. Eur Urol. 2020;77(5):628–635. 10.1016/j.eururo.2018.07.003 30041833

[8] Wagaskar VG , Mittal A , Sobotka S , Ratnani P , Lantz A , Falagario U , et al. Hood technique for robotic radical prostatectomy‐preserving periurethral anatomical structures in the space of Retzius and sparing the pouch of Douglas, enabling early return of continence without compromising surgical margin rates. Eur Urol. 2021;80(2):213–221. 10.1016/j.eururo.2020.09.044 33067016

[9] Shimmura H , Banno T , Nakamura K , Murayama A , Shigeta H , Sawano T , et al. A single‐center retrospective comparative analysis of urinary continence in robotic prostatectomy with a combination of umbilical ligament preservation and Hood technique. Int J Urol. 2023;30(10):889–895. 10.1111/iju.15227 37345368

[10] Galfano A , di D , Sozzi F , Strada E , Petralia G , Bramerio M , et al. Beyond the learning curve of the Retzius‐sparing approach for robot‐assisted laparoscopic radical prostatectomy: oncologic and functional results of the first 200 patients with ≥ 1 year of follow‐up. Eur Urol. 2013;64(6):974–980. 10.1016/j.eururo.2013.06.046 23856036

[11] Menon M , Dalela D , Jamil M , Diaz M , Tallman C , Abdollah F , et al. Functional recovery, oncologic outcomes and postoperative complications after robot‐assisted radical prostatectomy: an evidence‐based analysis comparing the Retzius sparing and standard approaches. J Urol. 2018;199(5):1210–1217. 10.1016/j.juro.2017.11.115 29225060

[12] Olivero A , Galfano A , Piccinelli M , Secco S , di Trapani D , Petralia G , et al. Retzius‐sparing robotic radical prostatectomy for surgeons in the learning curve: a propensity score‐matching analysis. Eur Urol Focus. 2021;7(4):772–778. 10.1016/j.euf.2020.03.002 32192919

[13] Hussein H , Maitra N , Tay J , Saxionis I , Makin R , Sivathasan S , et al. Analysis of the learning curve for Retzius‐sparing robot‐assisted radical prostatectomy for a single surgeon. J Clin Urol. 2023. 10.1177/20514158231203766

[14] Laird A , Fowler S , Good DW , Stewart G , Srinivasan V , Cahill D , et al. British Association of Urological Surgeons (BAUS). Contemporary practice and technique‐related outcomes for radical prostatectomy in the UK: a report of national outcomes. BJU Int. 2015;115(5):753–763. 10.1111/bju.12866 25046349

[15] Aning JJ , Reilly GS , Fowler S , Challacombe B , McGrath JS , Sooriakumaran P , et al. Perioperative and oncological outcomes of radical prostatectomy for high‐risk prostate cancer in the UK: an analysis of surgeon‐reported data. BJU Int. 2019;124(3):441–448. 10.1111/bju.14687 30681267

[16] Galfano A , Secco S , Dell'Oglio P , Rha K , Eden C , Fransis K , et al. Retzius‐sparing robot‐assisted radical prostatectomy: early learning curve experience in three continents. BJU Int. 2021;127(4):412–417. 10.1111/bju.15196 32745367

[17] Tay LJ , Makin R , Saxionis I , Dokubo I , Patel K , Sivathasan S , et al. Comparative analysis of early post‐operative outcomes between retzius‐sparing and anterior approach robotic radical prostatectomy for a single surgeon. J Clin Urol. 2023. 10.1177/20514158231156314

[18] Umari P , Eden C , Cahill D , Rizzo M , Eden D , Sooriakumaran P . Retzius‐sparing versus standard robot‐assisted radical prostatectomy: a comparative prospective study of nearly 500 patients. J Urol. 2021;205(3):780–790. 10.1097/JU.0000000000001435 33086025

[19] Gondoputro W , Thompson J , Evans M , Bolton D , Frydenberg M , Murphy D , et al. How does age affect urinary continence following robot‐assisted radical prostatectomy? A prospective multi‐institutional study using independently collected, validated questionnaires. J Urol. 2022;207(5):1048–1056. 10.1097/JU.0000000000002391 34978202

[20] Barakat B , Othman H , Gauger U , Wolff I , Hadaschik B , Rehme C . Retzius sparing radical prostatectomy versus robot‐assisted radical prostatectomy: which technique is more beneficial for prostate cancer patients (MASTER study)? A systematic review and meta‐analysis. Eur Urol Focus. 2022;8(4):1060–1071. 10.1016/j.euf.2021.08.003 34429272

[21] Dall CP , Mason JB , Choudhury E , Mora‐Garijo B , Egan J , Hu J , et al. Long‐term outcomes of pelvic‐fascia sparing robotic‐assisted radical prostatectomy versus standard technique: superior urinary function and quality of life without compromising oncologic efficacy in a single‐surgeon series. Urol Oncol. 2024;42(3):67.e17–67.e24. 10.1016/j.urolonc.2023.11.020 38212151

